# The health economics of social prescribing: systematic review of the international evidence

**DOI:** 10.3389/fpubh.2026.1753435

**Published:** 2026-01-28

**Authors:** Mary Lynch, Aoife Joan Keating, Elizabeth Morrow, Llinos Haf Spencer

**Affiliations:** 1 Faculty of Nursing and Midwifery, Royal College of Surgeons in Ireland, University of Medicine and Health Sciences, Dublin, Ireland; 2 Welsh Institute for Health and Social Care, Faculty of Life Sciences and Education, University of South Wales, Pontypridd, United Kingdom

**Keywords:** community referral, health economics, non-medical referral, social prescribing, Social Return on Investment (SROI)

## Abstract

**Background:**

Social prescribing is an approach to improving health and wellbeing that links individuals to community-based supports, such as arts programs, physical activity initiatives, financial or housing advice, volunteering opportunities, and social groups. Although evidence supporting its effects on physical, functional, and mental health outcomes is expanding, no systematic review of the international evidence has yet assessed the health economics of social prescribing.

**Aim:**

To systematically review evidence on the health economic methods and tools that have been used to evaluate social prescribing initiatives internationally.

**Methods:**

A systematic search of seven electronic databases (PubMed, Embase, Cochrane Trials, APA PsycINFO, CINAHL, Web of Science, and Ovid Global Health) was conducted alongside gray literature and citation searching. Two reviewers independently screened titles, abstracts, and full texts using Covidence, with disagreements resolved with a third researcher. Data extraction followed a structured protocol, and thematic analysis informed a narrative synthesis of findings.

**Results:**

Eighteen studies met the inclusion criteria: 5 randomized controlled trials, one quasi-experimental study, and 12 mixed-methods studies. Interventions reflected four main categories: exercise-based or loneliness-prevention initiatives (*n* = 10), coaching programs (*n* = 3), nature-based interventions (*n* = 3), and dance or movement-based programs (*n* = 2). Use of standard economic methods and tools was limited. Social Return on Investment analyses reported positive returns for interventions targeting mental health and loneliness.

**Conclusion:**

Robust economic evidence on social prescribing remains limited. Despite the availability of established health economic methods and tools, these are rarely applied to social prescribing, limiting the usefulness of existing studies for healthcare planning and commissioning.

## 1 Introduction

Social prescribing is an approach to improving health and wellbeing that links individuals to community-based supports, such as arts programs, physical activity initiatives, financial or housing advice, volunteering opportunities, and social groups (1). Although evidence supporting social prescribing's effects on physical, functional, and mental health outcomes is expanding, no systematic review of the international evidence has yet assessed the health economics of social prescribing (2). Thus, this systematic review article begins by outlining the theoretical, historical, and international foundations of social prescribing. It sets out the principles that have shaped social prescribing, before considering the role the health economics in social prescribing.

### 1.1 Theoretical, historical and international perspectives of social prescribing

Social prescribing has been defined as a healthcare approach that connects individuals, particularly those with chronic conditions, mental health challenges, or social needs, to non-clinical community-based services to assist and improve physical, functional, mental health and wellbeing (3, 4). These community assets may include arts groups, exercise programs, financial counselling, or volunteering opportunities, companionship or advice, as well as other health and wellbeing or supportive interventions (5–7). Within palliative and rehabilitative contexts, social prescribing offers a mechanism to enhance social participation, maintain function, and support quality of life, aligning closely with universal personalized care.

The term social prescribing originated in the United Kingdom in the 1970′s and gained prominence through the National Health Service Long Term Plan (8, 9). It was not until 2016, with the formation of the International Social Prescribing Network, that an official definition of social prescribing was established. This has since been developed through Delphi methods by the National Academy for Social Prescribing (10), as follows:

“A means for trusted individuals in clinical and community settings to identify that a person has non-medical, health-related social needs and to subsequently connect them to non-clinical supports and services within the community by co-producing a social prescription — a non-medical prescription, to improve health and wellbeing and to strengthen community connections.” (Page 4)

Worldwide, social prescribing terminology and definitions vary. Different terms such as community referral, non-medical referral, and link worker schemes are also commonly used to explain the process of connecting individuals to community-based programs and schemes (11). In Australia and New Zealand, the terms ‘cultural prescribing' or ‘community-led health programs', reflect initiatives that aim to incorporate indigenous knowledge in their design and delivery. In Canada and the United States of America (USA), related concepts include ‘patient navigation', ‘community health worker interventions', and ‘wellness linkages', typically embedded within broader public health or integrated care systems (11). All abbreviations can be found in Supplementary file 1.

A definition developed from the literature and applied in this review is:

Social prescribing, also known as community referral or cultural prescribing, refers to the structured process through which healthcare providers can connect individuals to non-medical, community-based support. Social prescribing operates on the principle that many factors influencing health and wellbeing, such as social isolation, reduced physical activity, or limited engagement in meaningful roles, fall outside the remit of conventional clinical care. Typically facilitated by a “link worker” or similar intermediary role, social prescribing enables patient access support including community groups, exercise programs, creative activities, peer-support networks, and practical advice (12).

In the UK National Health Service (NHS) the development of social prescribing has focused on the role of link workers and non-clinical staff who help patients access community-based support (13). Primary health care providers, such as GPs, refer patients to a link worker, who has a vital role in connecting patients with community resources and support services that best match the needs and circumstances of the individuals (14). In England, social prescribing is a formalized component of the primary care system, supported by dedicated link workers known as social prescribers. In Wales, the social prescribing model allows for self-referrals as well as referrals from healthcare professionals and social prescribers (15).

In Europe, social prescribing models link individuals to non-clinical community-based supports, but the structure, workforce, and referral processes differ. In contrast, in Finland and the Slovak Republic, social prescribing remains still in the pilot phase, and referrals are typically made by any healthcare professional involved in patient care. In the Republic of Ireland, the Health Service Executive (HSE) launched a national Social Prescribing Framework in 2021, which now supports the delivery of social prescribing services in over 30 locations across the island of Ireland (16).

As social prescribing gains traction internationally, it reflects broader movements toward person-centered, preventive, holistic and sustainable models of care (15). Social prescribing recognizes the multifaceted nature of health and the influence of social determinants of health such as access to health information, social experiences, and learning opportunities (17, 18). There has been a sharp increase in social prescribing programs globally, with many governments beginning to integrate social prescribing into their healthcare systems.

Social prescribing models draw on, and intersect with, broader concepts such as integrated care, which emphasizes coordinated delivery across health and social services, and asset-based community development (ABCD), a community-led approach that focuses on local strengths and participatory engagement. Together, these frameworks align with the core philosophies of social prescribing, including empowerment, inclusion, and the promotion of community resilience (19).

Sustainability is another principle that informs social prescribing. Evidence shows that social prescribing reduces the pressure on primary healthcare services and allows them to respond to a broader range of patient needs than traditional healthcare provision allows (20). Social prescribing approaches can have a significant impact on patients by providing them with a sense of community and connection. For example, social support services and group activities can promote overall wellbeing and social inclusion (21).

Guidance on social prescribing published by the World Health Organization in 2008 had a substantial influence on GPs and community-based services (22). This guidance helped formalize the rationale for linking patients to non-medical supports, emphasized the role of primary care in addressing social determinants of health, and encouraged the development of structured referral pathways to community resources. It also contributed to growing recognition of the need for integrated, person-centred approaches that extend beyond traditional clinical interventions. As a result, many primary care settings began to adopt or adapt social prescribing frameworks, laying the groundwork for more systematic implementation in subsequent years.

Different branches of social prescribing have emerged from different disciplines and traditions. For example, arts on prescription, and green or blue space prescribing, which involve engagement with arts and creative health activities or nature-based social prescribing interventions, respectively. Green prescribing involves activities such as walks, forest bathing, hikes and gardening, while blue prescribing involves time spent near bodies of water or swimming (23). Both arts-based/creative and nature-based approaches have been shown to improve cardiovascular health, immune response, depression, and anxiety symptoms (24).

The heterogeneous nature of social prescribing and its underpinning principles has given rise to several implementation challenges (25). These include variability in referral pathways, inconsistent definitions and delivery models, limited integration between healthcare and community sectors, and inadequate resourcing of link workers and community organizations. In addition, the absence of standardized outcome measures and robust evaluation frameworks makes it difficult to assess effectiveness and compare models across settings. Together, these challenges point to the need for clearer evidence-based operational frameworks, sustainable funding models, and stronger cross-sector collaboration to deliver effective and equitable social prescribing.

### 1.2 The role of health economics in social prescribing

Health economics is the study of how limited resources are allocated and utilized to improve population health and deliver healthcare efficiently, equitably, and effectively. It applies economic theories and methods to analyze the costs, benefits, and value of public health interventions, informing decisions about how best to organize, finance, and prioritize healthcare services. Through established approaches and methods such as cost-effectiveness, cost-utility, and cost-benefit analysis, health economics provides an evidence base to guide policymakers and practitioners in achieving maximum health gain from available resources (26–28).

Economic methods, such as cost benefit analysis (CBA), applying Social Return on Investment (SROI), Cost Effectiveness Analysis (CEA) and Cost Utility Analysis (CUA) are increasingly used to demonstrate the economic dimensions of healthcare (29). These methods could help healthcare systems and policymakers assess the value, cost-efficiency, and broader impacts of social prescribing on health and wellbeing outcomes. Among these, SROI has become the more widely used in recent years, as this structured method offers a robust framework for capturing the social, economic, and environmental value generated by social prescribing interventions (30).

Robust economic evaluations of social prescribing have only started to emerge in the past decade (8, 31, 32), which is the reason why this systematic review takes 2015 as the starting point for reviewing the literature. Research evaluation findings provides an assessment of the economic impact of social prescribing, synthesizing evidence on costs, savings, and system efficiencies across England (31). Produced for the National Academy for Social Prescribing, the report outlines how social prescribing generates value across health, social care, and community sectors and identifies key methodological challenges in measuring its full economic contribution. These challenges include short evaluation timeframes, small and heterogeneous program samples, reliance on self-reported or incomplete data, underestimation of community sector costs, and inconsistent or non-validated approaches to outcome measurement.

Despite a growing global evidence base supporting the benefits of social prescribing, and its increasing prominence in political discourse and social care practice, there are no previous systematic reviews examining the international evidence on the economic methods and tools applied in the evaluation of social prescribing initiatives. It may be that the diversity and complexity of social prescribing makes it difficult or unfeasible to apply traditional health economics approaches (8, 31–33). Capturing the true value of social prescribing may require broader, more flexible economic frameworks that can account for its cross-sector impacts and the holistic benefits it delivers to individuals and communities.

Previous authors have argued that it is essential that high-quality research trials assessing cost-effectiveness are conducted, enabling the evidence base to catch up with policy and helping to ensure that valuable time and resources are not wasted (34). Without a comprehensive understanding of the current usage of health economic evaluation in relation to social prescribing, both research and practice remain limited by insufficient robust evidence.

## 2 Aims

This systematic review aimed to examine evidence on the health economic methods and tools that have been used to evaluate social prescribing initiatives internationally. The primary research question was: What standard economic methods and tools have been used to evaluate social prescribing interventions for health and wellbeing internationally?

Four sub-questions guided the review:

1. Which types of health economic evaluations (e.g., cost-effectiveness, cost-utility, cost-benefit analysis, cost-consequence analyses) have been undertaken internationally and for what purposes?
2. What measurement tools, metrics, and outcomes are used to assess economic impact?
3. How are economic evaluations designed and implemented, including data sources, analytic frameworks, time horizons, perspectives, and methodological rigor?
4. What key evidence gaps, challenges, and opportunities exist for improving economic evaluation methods in future social prescribing research and practice?

## 3 Methods

### 3.1 Approach

Systematic review methodology provides a rigorous and transparent approach to identifying, appraising, and synthesizing evidence across multiple studies. By using predefined search strategies, inclusion criteria, and structured data extraction, systematic reviews minimize bias and allow for a comprehensive overview of existing research. This methodology is particularly well-suited for analyzing the use of economic methods in social prescribing, as it enables the systematic comparison of evaluation approaches, measurement tools, and study designs across diverse settings and contexts, while also highlighting gaps and inconsistencies in the current evidence base.

This article follows the Preferred Reporting Items for Systematic Reviews and Meta-Analyses (PRISMA) 2020 guidelines for the transparent and complete reporting of systematic reviews and meta-analyses. The guidelines comprise a 27-item checklist and a four-phase flow diagram designed to ensure systematic reviews clearly describe their methods and findings.

### 3.2 Protocol and registration

The review protocol was developed a priori to ensure transparency and methodological rigor. The systematic review protocol is available from: https://www.crd.york.ac.uk/PROSPERO/view/CRD420251142999.

### 3.3 Eligibility criteria

Peer-reviewed studies published between 2015 and 2025 (to November) were considered for inclusion. Eligible studies evaluated or described interventions related to social prescribing, peer support, exercise referral, health promotion, independent living, mental/emotional health, or community-based care models with a health economic component. Studies were excluded if they did not describe social prescribing interventions, focused solely on clinical, institutional, or biomedical models of care without an economic evaluation, or were commentaries, editorials, theses, or non-peer-reviewed literature. Although not all studies explicitly identified themselves as social prescribing interventions, those that did not self-designate were included when their content was deemed relevant to the scope of this review. Publications in languages other than English and those published prior to 2015 were also excluded.

### 3.4 Information sources

The electronic databases searched included PubMed, Embase, Cochrane Trials, APA PsycINFO, CINAHL, Web of Science, and Ovid Global Health. These databases were selected for their comprehensive international coverage of health, social care, and wellbeing literature, and for their combined breadth across clinical, psychological, public health, and interdisciplinary research domains. In addition to peer-reviewed sources, grey literature searches were conducted to capture relevant reports, policy documents, and evaluations that may not be indexed in academic databases. Citation tracking, both backward and forward, was also undertaken to identify additional studies that met the inclusion criteria and to ensure maximal coverage of the available evidence.

### 3.5 Search strategy

The PICO framework was utilized to develop the search strategy, incorporating population, intervention, comparison, and outcome keywords (see Table 1). Boolean operators and MeSH terms were combined to maximize sensitivity and specificity (35). The detailed PubMed search strategy is presented in Supplementary file 2.

**Table 1:** Population, Intervention, Comparison and Outcome (PICO) framework.

	**Inclusion criteria**	**Exclusion criteria**
Population	People taking part in social prescribing interventions	Studies that were not focused on social prescribing
Intervention	Social prescribing initiative with a health economic component	Studies that were not focused on social prescribing
Comparison	Usual care	N/A
Outcomes	Physical health outcomes Mental health outcomes Loneliness prevention	Any outcomes not related to health and wellbeing benefits of social prescribing
Language	English	Languages other than English
Dates	2015–2025	Pre-2015

### 3.6 Study selection

Titles and abstracts identified through database searches were independently screened by two reviewers. Full-text articles were then assessed for eligibility. Disagreements were resolved through discussion or consultation. The selection process is summarized in a PRISMA flow diagram.

### 3.7 Inclusion criteria

Peer reviewed studies published between 2015 and November 2025, which evaluated or described interventions related to social prescribing, peer support, exercise referral, health promotion, independent living, mental/emotional health, or community-based support models, with a health economic component, were included in this systematic review.

### 3.8 Exclusion criteria

Peer reviewed articles that do not describe social prescribing interventions, or that focus on clinical, institutional, or biomedical models of care without a health economic component were excluded. Commentaries, editorials, and theses, were not included in this systematic review. Studies in languages other than English were excluded, as were articles published before 2020.

During the screening process the exclusion criteria were further refined through discussions between team members regarding scope and the definition of social prescribing (LHS, AJK, EM). Key decisions were as follows. The review excluded studies involving community initiatives for people with serious or severe mental health conditions (e.g., psychosis) as this level of support is clinical needs. Broader social care interventions, such as community farm residency programs for individuals with learning disabilities or mental health conditions were excluded. These interventions were considered to fall outside the scope of social prescribing and more appropriately classified as community-based social care. Similarly, initiatives delivered within assisted living or residential settings were excluded on the basis that they constituted social care rather than social prescribing models. Approaches using telephone-only supportive interventions were excluded as well, as they did not involve group-based or community-centred components which are central to the definition of social prescribing (see below).

### 3.9 Data extraction

Data were extracted using a standardized form. Extracted items included study characteristics (author, year, country, study design), intervention details (type, duration, target population), economic evaluation type (cost-effectiveness, cost-utility, cost-benefit, cost-consequence), measurement tools and outcomes (QALY, DALY, HRQoL instruments, cost metrics), and the perspective of economic analysis (societal, healthcare system).

### 3.10 Quality assessment

Methodological quality was assessed using JBI critical appraisal tools (36–38). The Consolidated Health Economic Evaluation Reporting Standards (CHEERS) 2022 checklist for economic evaluations was not used as most of the studies did not report on full economic evaluations and focused mainly on SROI studies (39). Studies were evaluated on reporting clarity, methodological rigor, and transparency. Although all studies were assessed for quality, no studies were excluded based on low quality. Quality appraisal tables can be found in Supplementary file 3.

### 3.11 Data synthesis

A narrative synthesis was performed due to anticipated heterogeneity in interventions, study designs, and economic evaluation methods (40). Data were summarized in tables and text to describe types of economic evaluation, outcomes measured, tools used, and methodological quality. Where relevant, comparisons between countries or categories of social prescribing intervention types were explored.

### 3.12 Reporting and transparency

This review adheres to the PRISMA 2020 checklist for systematic reviews. The search strategy for PubMed was provided in Supplementary file 1.

### 3.13 Ethical statement

Ethical approval was not required for this systematic review as it involved the analysis of publicly available data and did not include any direct interaction with human participants.

## 4 Results

In accordance with PRISMA systematic review reporting guidelines, the results are presented first as an overview of the included literature, followed by an analysis of key themes and evidence gaps, structured to address the research questions (see Aims).

### 4.1 Overview of the included literature

**Figure 1:**
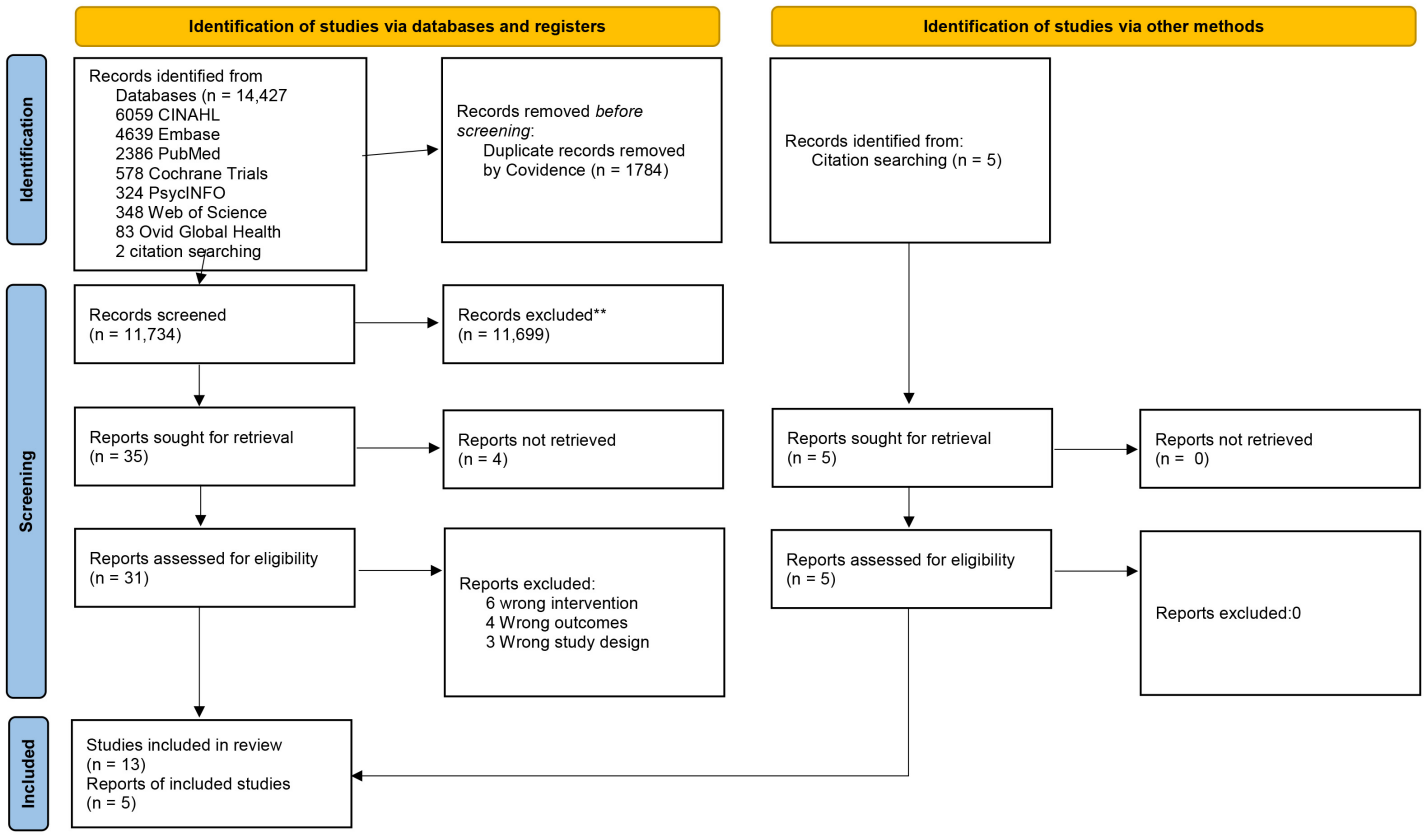
PRISMA 2020 flow diagram for the Health Economics and Social Prescribing (HESP) systematic review. Consider, if feasible to do so, reporting the number of records identified from each database or register searched (rather than the total number across all databases/registers). **If automation tools were used, indicate how many records were excluded by a human and how many were excluded by automation tools. **Source** Page et al. (41). This work is licensed under CC BY 4.0. To view a copy of this license, visit https://creativecommons.org/licenses/by/4.0/.

The PRISMA 2020 flow diagram for this review is presented in Figure 1. A total of 14,427 articles were identified and imported into the Covidence software platform for review. Following removal of duplicates 11,734 titles were screened based on title/abstract. Of these 35 studies were selected for full text review. Eighteen studies met the inclusion criteria and were included in the review. Reasons for exclusion of the other 17 articles are shown in Figure 1.

Of the 18 included studies, five were randomized controlled trials (RCTs), one quasi-experimental study, and twelve mixed-methods studies. Interventions fell into four main categories: exercise-based or loneliness-prevention initiatives (*n* = 10), coaching programs (*n* = 3), nature-based interventions (*n* = 3), and dance or movement-based programs (*n* = 2) (detail below). SROI analyses consistently reported positive returns, particularly for interventions targeting mental health and loneliness. Evidence from randomized trials was mixed regarding quality-of-life outcomes and participant satisfaction.

An overview of all included studies (*n* = 18) is presented in Table 2.

**Table 2:** Overview of included studies study design and intervention type.

**Type of intervention**	**Exercise based or loneliness prevention social prescribing**	**Nature based social prescribing**	**Coaching based social prescribing**	**Dance and movement based social prescribing**	**Number of studies**
Randomized controlled trial (RCT)	Coulton et al. (42); Deidda et al. (43); Ellis-Hill et al. (44)	–	–	Clifford et al. (45); Tew et al. (46)	5
Quasi-experimental study	Galbraith et al. (47)	–	–	–	1
Mixed methods study	Foster et al. (48); Gandy et al. (49); Hartfiel et al. (50); Jones et al. (51); Whiteley et al. (52); Willis et al. (53)	Lynch et al. (54); Makanjuola et al. (55); Makanjuola et al. (56)	Makanjuola et al. (57); Moffatt et al. (58); Skinner et al. (59)	–	12
Total	10	3	3	2	*N =* 18 papers Including 13 from database searches; *N =* 5 from citation lists

Table 3 synthesizes the economic methods and tools used in each study.

**Table 3:** Economic methods and tools used in the included social prescribing studies.

**Author and date**	**Country**	**Type of intervention**	**Main health economic method**	**Outcome measurement tools**	**Economic outcomes**
Clifford et al. (45)	Ireland	‘Music and Movement for Health (MMH)' intervention	Cost-effectiveness feasibility	EuroQol-5 Dimensions, five-level version (EQ-5D-5L)	The MMH intervention is likely to be cost-effective (feasibly study only).
Coulton et al. (42)	England	Community singing	Cost-effectiveness	SF12; Hospital Anxiety and Depression Scale; EQ-5D	The community singing intervention was cost-effective, p = 0.015 (95% CI 0.014–0.016).
Deidda et al. (43)	Northern Ireland; Spain; Germany; Denmark	Exercise referral and Exercise referral with self-management strategies.	Cost-effectiveness	EQ-5D-5L; ICECAP-O; Tailored cost logs	Exercise with self-management wis more cost-effective than exercise referral alone.
Ellis-Hill et al. (44)	England	Community-based arts and health intervention	Cost consequence	Warwick–Edinburgh Mental Wellbeing Scale (WEMWBS); ICEpop CAPability measure for adults (ICECAP-A); Rosenberg Self-Esteem Scale (RSES); Medical Outcomes Short Form Health Survey (SF-36 V.1)	The community arts and health-based intervention for stroke survivors cost an estimated £456 per person. A full cost-effectiveness evaluation was not conducted.
Foster et al. (48)	United Kingdom	British Red Cross funded link-workers	SROI	UCLA Loneliness Scale; Short Warwick–Edinburgh Mental Wellbeing Scale (SWEMWBS)	For each £1 spent on the program, there was an estimated return on investment of £3.42. This reflected improvements in wellbeing, reduced loneliness, and other associated benefits.
Galbraith et al. (47)	England	Active HERE physical activity intervention.	Cost-utility and cost-effectiveness.	International Physical activity Questionnaire Short Form (IPAQ-S); Single Item Sport England Measure (SISEM); New General Self-Efficacy Scale (NGSE); Five-item World Health Organization Wellbeing Index (WHO-5).	Both intervention pathways were cost-effective and cost-saving for participants aged ≥61 years over a short time horizon, with the Motivational Interviewing pathway yielding higher return on investment (ROI) estimates.
Gandy et al. (49)	England	Active Lives Program	Cost benefit	Custom designed outcome indicators	The overall average cost per active lives program session was £81.09 in 2017, and the overall average cost per person was £160.67. The overall average cost per attendance was £2.34.
Hartfiel et al. (50)	Wales	Coed Lleol—Small Woods woodland activity intervention.	SROI	SWEMWBS; 7-day physical activity recall; General self-efficacy scale social trust question	Social values of between £2.57 and £4.67 for every £1 invested were found with improvements in physical activity, self-efficacy and social trust for those who took part in the active woodland interventions.
Jones et al. (51)	Wales	Health precinct study	SROI	EQ-5D; Rosenberg Self-Esteem Scale; Campaign to end Loneliness Scale	The health precinct study for people with chronic health conditions demonstrated a base case social value of £5.07 for every £1 invested in the health precinct led by various agencies in north Wales.
Lynch et al. (54)	Wales	‘Making Well' A Nature Based Social prescribing program	SROI	The measurement tools were not named in the abstract.	The Fathom Trust ‘Making Well' intervention for people with chronic mental health conditions demonstrated a positive social value (£0.30 to £4.70 for every £1 invested).
Makanjuola et al. (57)	Wales	Emotional Mind Dynamic intervention	SROI	SWEMWBS; General Self-Efficacy Scale (GSES)	The EMD coaching SROI demonstrated social values ranging from £4.12–£7.08 for face-to-face clients compared with £2.37–£3.35 for online participants.
Makanjuola et al. (55)	Wales	Opening the doors to the outdoor (ODO) partnership	SROI	SWEMWBS; A social trust question, an overall health question; The International Physical Activity Questionnaire- short form.	The ODO partnership program demonstrated a social value of between £4.90 and £5.36 per £1 invested.
Makanjuola et al. (56)	Wales	Wrexham University Nature Based Social Prescribing	SROI	SWEMWBS; General Self-Efficacy Scale (GSES)	For each £1 invested, there was a positive social value of between £1.83 and £2.38.
Moffatt et al. (58)	England	The SPRING_NE intervention for adults with type 2 diabetes	Cost-consequence analysis (CCA) approach	EQ-5D-5L	Outcome effects varied across different groups, and the experience of social prescribing differed depending on client circumstances.
Skinner et al. (59)	Wales	MY LIFE programme	SROI	SWEMWBS; EQ-5D-5L	The SROI demonstrated that the estimated social value for every £1 invested for participants who engaged with the ‘Diabetes Technician only' ranged from £4.67 to £4.70. The social value for participants who engaged with the ‘Diabetes Technician and social prescribing program' ranged from £4.23 to £5.07.
Tew et al. (46)	England and Wales	Chair-based yoga intervention	Cost-effectiveness analysis	EQ-5D-5L; PROMIS-29	Authors stated that the intervention was cost-effective, however, there were no HRQoL improvements.
Whiteley et al. (52)	Wales	Volunteering at a Botanic Garden in North Wales	SROI	SWEMWBS; The ICECAP-A; The Basic Psychological Need Satisfaction and Frustration Scale (BPNSNF); The Nature Connection Index (NCI)	A positive return on investment of £4.02 to £5.43 for every £1 invested was found for the volunteer gardening program. There were significant wellbeing benefits and associated social value to local communities, including a reduced burden on overstretched local healthcare services
Willis et al. (53)	England	Peer support for people with dementia	SROI	Custom outcome indicators developed through stakeholder engagement to capture changes in: Mental stimulation Reduction in loneliness and isolation Improved wellbeing Reduced carer stress and burden Increased knowledge of dementia for volunteers.	For every £1 invested in the peer support program for people with dementia, the SROI ranged from £1.17 to £5.18 depending on group structure.

### 4.2 Types and purposes of economic evaluations in social prescribing

This part of the results presents the economic methods used according to different types of social prescribing interventions in order to show how approaches vary by intervention type. Grouping the included studies into four categories—exercise-based or loneliness-prevention (*n* = 10), coaching (*n* = 3), nature-based (*n* = 3), and dance or movement-based programs (*n* = 2)—makes it possible to compare how economic analyses are applied across these different types of social prescribing.

Exercise-based or loneliness-prevention social prescribing interventions featured most prominently in the included literature (*n* = 10) (42–44, 47–53). These studies included economic evaluations, with three RCTs, one quasi-experimental study, and six mixed-methods SROI studies (Table 2) For example, the SITLESS program 47 combined exercise referral schemes with self-management strategies across four European countries, demonstrating cost-effectiveness and increased physical activity (43). Galbraith et al. (47) evaluated the ACTIVE intervention and found motivational interviewing pathways to be cost-effective and cost-saving (47). SROI studies, such as Jones et al. (51), Hartfiel et al. (50), Whiteley et al. (52), and Foster et al. (48), consistently showed positive returns for exercise-based and loneliness-prevention interventions (48, 50–52).

Nature-based social prescribing (NBSP) was evaluated in three mixed-methods SROI studies (52, 55, 56), with interventions including outdoor walking, climbing, and gardening. SROI ratios ranged from £1.83 to £5.36 per £1 invested, with improvements in wellbeing, social connection, and physical activity.

Studies of coaching-based interventions (*n* = 3) included the Emotion Mind Dynamic (EMD) program (57), which combined coaching, mentoring, and mindfulness. For face-to-face participants, SROI ranged from £4.12–£7.08 per £1 invested, and for online participants £2.37–£3.35. Two T2DM-targeted programs (58, 59) demonstrated mixed outcomes, with the MY LIFE program showing higher social value when patients engaged with both the diabetes technician and social prescribing pathways.

Two studies evaluating dance and movement-based social prescribing incorporated economic evaluations to assess feasibility of the intervention and potential cost-effectiveness. Clifford et al. (45) evaluated the Music and Movement for Health (MMH) program for older adults in Ireland using an RCT. The EQ-5D-5L tool measured health-related quality of life (HRQoL), showing improvements in psychosocial measures for participants, and the economic analysis demonstrated feasibility for future cost-effectiveness studies (45). The health economic analysis confirmed the feasibility of the methodology employed and points to the potential cost-effectiveness of the MMH intervention relative to the control or no organized program. Tew et al. (46) evaluated chair-based yoga for older adults with multimorbidity across 15 GP practices in the UK. The intervention cost £80.85 more per participant but generated an additional 0.0178 QALYs, with a 79% probability of being cost-effective at a £20,000 willingness-to-pay threshold (46). However, no statistically significant improvements in HRQoL, mental health, or loneliness were observed. The intervention was generally perceived as safe, deemed acceptable by many participants, and regarded as highly beneficial by a subset of individuals.

Of the 18 included studies, ten used SROI to evaluate social prescribing interventions (see Table 3). These included economic analysis of link-worker programs, physical activity programs, nature-based activities, coaching, volunteer gardening, and dementia peer support (23, 48–50, 52, 53, 55–57, 59). Target populations included older adults, people with chronic conditions, those with mental health challenges, students, volunteers, and adults at risk of type 2 diabetes. SROI ratios were consistently positive, ranging from £1.17 to £7.08 per £1 invested, with benefits including improved wellbeing, social connectedness, self-efficacy, and reductions in loneliness or isolation. These findings demonstrate the potential for social prescribing interventions to generate substantial social and economic value for individuals and communities.

Traditional economic evaluation methods were also applied in the included studies: cost-effectiveness analysis (42, 45, 46), cost-utility analysis or combined cost-effectiveness and cost-utility (43, 47), cost-consequence analysis (44, 58), cost-benefit analysis (49).

### 4.3 Measurement tools, metrics, and outcomes used

A wide variety of measurement tools were employed across the 18 included studies (see Table 2). Health related quality of life (HRQoL) was commonly measured using the EQ-5D-5L, while mental health and wellbeing outcomes were captured using the Short Warwick–Edinburgh Mental Wellbeing Scale (SWEMWBS) and ICECAP measures. Physical activity was assessed via the International Physical Activity Questionnaire Short Form (IPAQ-SF). Client service receipt inventories (CSRI) were used to estimate costs, which varied across studies to include staff time, volunteer expenses, venue hire, and materials (52, 58).

Two SROI studies integrated Contingent Valuation (CV) methods to estimate participants' willingness to pay (WTP) (56, 57). These studies demonstrated the additional value individuals place on accessing social prescribing interventions. For example, WTP for the EMD program ranged from £600–£730 per participant (57), while students in the Wrexham University NBSP intervention were willing to pay £7 per week for access to good quality greenspace (56).

### 4.4 Design and implementation of economic evaluations

Economic evaluations varied in design, ranging from RCT-based cost-effectiveness analyses to mixed-methods SROI studies. Study designs included randomized allocation, quasi-experimental pre-post designs, and observational approaches (see Table 2). Hence, methodological rigor varied, with feasibility and pilot studies focusing on implementation and data collection, while larger RCTs included robust economic modeling. As previously described, analytic frameworks applied in these studies included cost-effectiveness analysis, cost-utility analysis, cost-benefit analysis, cost-consequence analysis, and SROI (see Table 3).

As anticipated, most studies adopted the perspective of the health and social care system, focusing on demonstrating the feasibility, efficiency, or cost-effectiveness of social prescribing interventions for healthcare providers and payers. This perspective typically considered direct costs such as program delivery, staff time, and healthcare utilization. Some studies (predominantly SROI) took a societal perspective, capturing a wider range of economic and social benefits, including impacts on wellbeing and social connectedness of participants, volunteer contributions, and broader community value (2, 42, 43, 45–53, 55, 56, 58, 59).

Time horizons for economic evaluations varied considerably across studies, ranging from the immediate duration of the intervention, typically weeks or months, to longer-term projections spanning several years. For example, Deidda et al. (43) developed a Markov decision-analytic model to project the long-term cost utility and capability outcomes of their combined exercise referral and self-management intervention over 5- and 15-year time horizons (43). Other studies adopted more conservative time frames focused on within-trial data, limiting their horizon to the duration of the program (45, 47).

### 4.5 Evidence gaps, challenges, and opportunities for future research

Across the 18 included studies, several gaps and challenges were identified:

- Limited use of standardized economic evaluation methods and outcome measures, hindering comparability across studies.
- Small sample sizes and feasibility designs in RCTs, limiting statistical power to detect differences in HRQoL or clinical outcomes.
- Variability in intervention design, delivery, and intensity, making replication and benchmarking difficult.
- Underrepresentation of certain populations and limited evidence in diverse geographical or socio-economic contexts.
- Sparse integration of patient-reported outcome measures (PROMs) and broader societal outcomes in evaluations (e.g., routes into education or employment outcomes).

Opportunities for future research include the development of standardized economic frameworks, expanded use of Contingent Valuation method and SROI methodology to capture social value, choice and preferences, longer-term follow-up, and improved integration of cost and outcome data to inform policy and commissioning decisions (33). These findings support previous calls for further research to identify who is most likely to benefit from social prescribing and what type of intervention is most cost effective (8, 31, 32).

## 5 Discussion

This systematic review has found that despite international developments in social prescribing and growing evidence of beneficial health and wellbeing outcomes, the current economic evidence for social prescribing remains limited. Few cost-effectiveness, cost-utility, or cost-benefit analysis studies have been conducted to date on different types of social prescribing initiatives. This lack of rigorous economic evaluation constrains the ability to draw firm conclusions about the costs or value for money of social prescribing initiatives. Consequently, there is limited economic evidence available for policymakers and commissioners to inform decisions about investment or effective and sustainable funding models (60).

Corroborating the findings of previous reviews, there is substantial heterogeneity in the economic outcome measures and metrics used across studies. While some report cost-effectiveness, others rely solely on cost data or service utilization (44, 45, 47). This lack of standardized and comparable metrics limits the ability to synthesize findings through meta-analysis and constrains efforts to benchmark interventions against one another or against alternative healthcare investments.

Methodological limitations are common in emerging literature on social prescribing (63). Many studies are small-scale, single-site, or non-randomized, with short follow-up periods that prevent assessment of long-term economic outcomes. Additionally, reporting on the perspective of analysis, whether societal or healthcare system may be inconsistent with patient outcomes or what matters to participants in these initiatives. Assumptions underlying analytical models, such as HRQoL are frequently underreported, limiting transparency and reproducibility of evaluation approaches (61).

Geographically, the economic evidence on social prescribing is largely concentrated in the UK (9 of 18 studies), with few studies from other countries or diverse populations. This limits the generalizability of findings and leaves gaps in understanding how social prescribing interventions function in different cultural, healthcare, or community contexts. Furthermore, mixed method approaches integrating economic evaluation with qualitative insights on participant and provider experiences are rare, despite their potential to enrich understanding of value and implementation challenges (62).

The identified studies, which report on economic considerations, typically provide incomplete information on cost components, resource utilization, or outcome valuation methods, reducing transparency and limiting the utility of the findings for informing policy or practice. Collectively, these gaps highlight the need for standardized, rigorous, and internationally diverse research to strengthen the evidence base on the health economics of social prescribing.

## 6 Strengths and limitations

The main strength of this review is that a wide range of databases were systematically searched using a comprehensive set of search terms to capture relevant literature across disciplines and countries. This approach maximized the potential for identifying diverse studies, including those from the UK and Europe, and helped mitigate publication bias.

The review followed recognized methodological PRISMA guidelines, made use of Covidence systematic review software, and included double data extraction and rigor procedures aligned with Cochrane standards. This enhanced the reliability and reproducibility of the findings. All included studies underwent structured quality assessment, allowing for a transparent evaluation of methodological robustness. This process enabled the identification of both high and lower-quality studies, contributing to a nuanced synthesis of the overall body of evidence on the topic.

The review was limited to studies published between 2015 and September 2025. While this timeframe ensured alignment with recent developments in social prescribing and economic evaluation, it may have excluded earlier foundational work that could offer valuable conceptual or methodological insights.

Although the review sought to capture evidence from a global perspective, the available literature was disproportionately concentrated in a small number of countries. In particular, there was a notable scarcity of studies from continental Europe and other world regions, which may limit the broader generalizability and transferability of the findings. Furthermore, despite the use of a comprehensive and systematic search strategy, the overall volume of peer-reviewed research explicitly examining the intersection of health economics and social prescribing remains limited. These limitations constrained the depth of analysis and reflects the relative novelty or underdevelopment of this field of inquiry.

## 7 Implications for future development

Drawing on these findings, five core recommendations are outlined to guide the future development of economic research in social prescribing:

*Recommendation (1) Reach of economic research:* Future research should seek to broaden the geographic reach of economic research in social prescribing, particularly in regions that remain underrepresented in the current evidence base, such as continental Europe and low- and middle-income countries. Expanding the global reach of this work is essential for improving the applicability, cultural relevance, and equity of economic evidence on social prescribing across diverse health and community settings.

*Recommendation (2) Long-term and comparative economic methods:* To strengthen understanding of the cost-effectiveness, social value, and long-term impact of social prescribing, future studies should prioritize longitudinal designs and comparative analyses across different health systems, populations, and policy environments. Such approaches would enable more robust assessments of sustainability, scalability, and value for money, thereby supporting better-informed policy and funding decisions.

*Recommendation (3) Standardized economic methods and measures:* To advance the field more broadly, several methodological and strategic priorities should be adopted. First, there is a need to standardize economic evaluation frameworks and outcome measures, including consistent use of cost-effectiveness analysis (CEA), cost-utility analysis (CUA), and social cost-benefit analysis (SCBA), as well as validated metrics such as QALYs, DALYs, and HRQoL instruments. Greater standardization would enable comparability across social prescribing studies and facilitate meta-analytic synthesis within categories of similar intervention types. Second, enhancing methodological rigor is crucial. Larger, multi-site, and longer-term evaluations, conducted with transparent analytic assumptions, clearly stated perspectives, and adherence to recognized reporting standards, would substantially improve the quality and credibility of the evidence base.

*Recommendation (4) Integrated evaluation:* Integrating mixed-methods approaches that combine economic evaluation with qualitative, participative or implementation research, for example SCBA applying SROI facilitates capturing costs to benefits along with monetary terms. SROI mixed method analysis provides the narratives on inputs, outputs and outcomes by means of development of a Theory of Change approach would provide richer insights into the mechanisms of impact, contextual drivers of effectiveness, and the lived experiences of service users and providers.

*Recommendation (5) Economic-informed policy decisions:* Strengthening the economic evidence base will enable policymakers and commissioners to make more strategic decisions regarding resource allocation, prioritizing interventions with demonstrable value and supporting the sustainable scaling of social prescribing initiatives. However, this requires research to policy knowledge to action frameworks to be developed to support policy implementation.

Together, these recommendations offer a foundation for improving the validity, relevance, and policy utility of future economic research in social prescribing.

## 8 Conclusions

This systematic review demonstrates the role and value of health economic methods and tools in the evaluation of social prescribing interventions internationally. Integration of health economics evaluation approaches can demonstrate the CEA, CUA, and SROI of social prescribing public health interventions and return on investment. However, very few social prescribing studies have included robust economic methods, which suggests that the integration of economic analysis into social prescribing research remains an emergent and underdeveloped area globally. The selection of appropriate health economic methods and tools is inherently contingent upon the awareness of the importance of economic considerations, the specific research questions asked, and the type of social prescribing intervention being addressed. This variation necessitates a tailored approach to evaluation design, integration of economic methods and knowledge to action frameworks to maximize policy utility.

The current methodological gap in the health economics of social prescribing presents a significant barrier to the generation of robust evidence capable of informing policy formulation, resource allocation and assessment of economic dimensions of healthcare sustainability. Consequently, there is an urgent need for the development and application of consistent, high-quality economic evaluation frameworks to substantiate the value and sustainability of social prescribing interventions in diverse populations and international contexts.

## Data Availability

Publicly available datasets were analyzed in this study. This data can be found here: llinosspencer@rcsi.ie.
