# Supplementary material for: The health economics of social prescribing: systematic review of the international evidence
Source: Front Public Health. 2026 Jan 28;14:1753435. doi: 10.3389/fpubh.2026.1753435 (PMC12891220; doi:10.3389/fpubh.2026.1753435)
Supplement: Supplementary file 1 [file Supplementary_file_1.docx]

## Supplementary File 1. List of abbreviations

| **Abbreviation** | **Full phrase** |
| --- | --- |
| ABCD | Assets-based Community Development |
| BPNSNF | Basic Psychological Need Satisfaction and Frustration Scale |
| CBA | Cost Benefit Analysis |
| CBC | Community Based Care |
| CEA | Cost Effectiveness Analysis |
| CENTRAL | Cochrane Central Register of Controlled Trials (database) |
| CINAHL | Cumulative Index to Nursing and Allied Health Literature (database) |
| CSRI | Client Service Receipt Inventory |
| CUA | Cost Utility Analysis |
| DALY | Disability Adjusted Life Year |
| DCE | Discrete Choice Experiment |
| DT | Diabetes Technician |
| Embase | Embase via Ovid (database) |
| ERS | Exercise Referral Scheme |
| GPs | General Practitioners |
| GYY | Gentle Years Yoga |
| HbA1c | Glycated Haemoglobin |
| HoS | HeART of Stroke |
| HRQoL | Health Related Quality of Life |
| IPAQ-S | International Physical Activity Questionnaire Short Form |
| MI | Motivational Interviewing |
| MMH | Music and Movement for Health |
| NBSP | Nature Based Social Prescribing |
| NCI | Nature Connection Index |
| NHS | National Health Service |
| NGSE | New General Self-Efficacy Scale |
| ODO | Opening Doors to the Outdoors |
| PA | Physical activity |
| PROMs | Patient-reported outcome measures |
| QALY | Quality Adjusted Life Year |
| QoL | Quality of Life |
| RCT | Randomized Control Trial |
| RCSI | Royal College of Surgeons in Ireland |
| ROI | Return on Investment |
| SISEM | Single Item Sport England Measure |
| SROI | Social Return on Investment |
| SMS | Self-management Scheme |
| TAU | Treatment as Usual |
| T2DM | Type 2 Diabetes Mellitus |
| UC | Usual Care |
| UCLA | University of California, Los Angeles |
| UK | United Kingdom |
| USA | United States of America |
| WHO-5 | five-item World Health Organization Well-Being Index |
| YFC | Years in full capacity |
