# Supplementary material for: The health economics of social prescribing: systematic review of the international evidence
Source: Front Public Health. 2026 Jan 28;14:1753435. doi: 10.3389/fpubh.2026.1753435 (PMC12891220; doi:10.3389/fpubh.2026.1753435)
Supplement: Supplementary file 2 [file Supplementary_file_2.docx]

## Supplementary File 2: Search strategy for PubMed

PubMed Search Strategy: Health Economics Social Prescribing 15-09-2025

| ***No.*** | ***Query*** | ***Last Run Via*** | ***Results*** |  |
| --- | --- | --- | --- | --- |
| #1 | "Community Health Planning"[Mesh] OR "Community Networks"[Mesh] OR "Community Health Services"[Mesh] OR "Nurses, Community Health"[Mesh] OR "Health Promotion"[Mesh] OR "Independent Living"[Mesh] OR "Home Care Services, Hospital Based"[Mesh] OR "care closer to home" OR "CBC"[Title] OR "community based care" OR "community connect" OR "community-based care" OR "exercise referral" OR "health promotion" OR "home from hospital" OR "independent living" OR "peer support" OR "promoting health" OR "social prescribing intervention*" | PubMed | 530,520 |  |
| #2 | "Anxiety"[Mesh] OR "Bipolar Disorder"[Mesh] OR "Mental Disorders"[Mesh:NoExp] OR "Panic Disorder"[Mesh] OR "Psychotic Disorders"[Mesh] OR "Obsessive-Compulsive Disorder"[Mesh] OR "Psychological Well-Being"[Mesh] OR "Anxiety" OR "Bipolar disorder*" OR "Chronic mental health problem*" OR "common mental health problem*" OR "Obsessive Compulsive Disorder*" OR "Panic attack*" OR "Psychosis" OR "serious mental health problem*" | PubMed | 683,197 |  |
| #3 | "Cost-Benefit Analysis"[Mesh] OR "Cost-Effectiveness Analysis"[Mesh] OR "Disability-Adjusted Life Years"[Mesh] OR "Health Impact Assessment"[Mesh] OR "Quality of Life"[Mesh] OR "Qualitative Research"[Mesh] OR "Quality-Adjusted Life Years"[Mesh] OR "benefit-cost ratio" OR "conjoint analysis" OR "contingent valuation" OR "cost outcome" OR "cost utility" OR "cost-benefit analysis" OR "cost-effectiveness" OR "DALY"[Title] OR "DCE"[Title] OR "Disability Adjusted Life Year" OR "discrete choice experiment*" OR "economic analysis" OR "economic benefit*" OR "economic evaluation" OR "economic saving*" OR "health impact assessment*" OR "health related quality of life" OR "impact analysis" OR "mixed methods stud*" OR "opportunity cost*" OR "QALY"[Title] OR "qualitative studies" OR "Quality Adjusted Life Year" OR "quantitative" OR "return on investment*" OR "revealed preference*" OR "social cost benefit*" OR "social return on investment*" OR "stated preference*" OR "trade-off" OR "travel cost model*" OR "willingness to pay" | PubMed | 1,562,983 |  |
| #4 | #1 AND #2 AND #3 | PubMed | 4,276 |  |
| #5 | #4 NOT ("Editorial" [Publication Type] OR "Comment" [Publication Type] OR "Clinical Trial Protocol" [Publication Type] OR "Edit*"[Title] OR "Comment*"[Title] OR "Protocol*"[Title]) | PubMed | 3,993 |  |
| #6 | #5 AND ("2020/01/01"[Date - Create] : "3000"[Date - Create]) | PubMed | 1,392 |  |
| #7 | #6 AND (english[Filter]) | PubMed | 1,362 |  |
|  | (((("Community Health Planning"[MeSH Terms] OR "Community Networks"[MeSH Terms] OR "Community Health Services"[MeSH Terms] OR "nurses, community health"[MeSH Terms] OR "Health Promotion"[MeSH Terms] OR "Independent Living"[MeSH Terms] OR "home care services, hospital based"[MeSH Terms] OR "care closer to home"[All Fields] OR "CBC"[Title] OR "community-based care"[All Fields] OR "community connect"[All Fields] OR "community-based care"[All Fields] OR "exercise referral"[All Fields] OR "Health Promotion"[All Fields] OR "home from hospital"[All Fields] OR "Independent Living"[All Fields] OR "peer support"[All Fields] OR "promoting health"[All Fields] OR "social prescribing intervention*"[All Fields]) AND ("Anxiety"[MeSH Terms] OR "Bipolar Disorder"[MeSH Terms] OR "Mental Disorders"[MeSH Terms:noexp] OR "Panic Disorder"[MeSH Terms] OR "Psychotic Disorders"[MeSH Terms] OR "Obsessive-Compulsive Disorder"[MeSH Terms] OR "Psychological Well-Being"[MeSH Terms] OR "Anxiety"[All Fields] OR "bipolar disorder*"[All Fields] OR "chronic mental health problem*"[All Fields] OR "common mental health problem*"[All Fields] OR "obsessive compulsive disorder*"[All Fields] OR "panic attack*"[All Fields] OR "Psychosis"[All Fields] OR "serious mental health problem*"[All Fields]) AND ("cost benefit analysis"[MeSH Terms] OR "Cost-Effectiveness Analysis"[MeSH Terms] OR "Disability-Adjusted Life Years"[MeSH Terms] OR "Health Impact Assessment"[MeSH Terms] OR "Quality of Life"[MeSH Terms] OR "Qualitative Research"[MeSH Terms] OR "Quality-Adjusted Life Years"[MeSH Terms] OR "benefit-cost ratio"[All Fields] OR "conjoint analysis"[All Fields] OR "contingent valuation"[All Fields] OR "cost outcome"[All Fields] OR "cost utility"[All Fields] OR "cost benefit analysis"[All Fields] OR "cost-effectiveness"[All Fields] OR "DALY"[Title] OR "DCE"[Title] OR "Disability Adjusted Life Year"[All Fields] OR "discrete choice experiment*"[All Fields] OR "economic analysis"[All Fields] OR "economic benefit*"[All Fields] OR "economic evaluation"[All Fields] OR "economic saving*"[All Fields] OR "health impact assessment*"[All Fields] OR "health related quality of life"[All Fields] OR "impact analysis"[All Fields] OR "mixed methods stud*"[All Fields] OR "opportunity cost*"[All Fields] OR "QALY"[Title] OR "qualitative studies"[All Fields] OR "Quality Adjusted Life Year"[All Fields] OR "quantitative"[All Fields] OR "return on investment*"[All Fields] OR "revealed preference*"[All Fields] OR "social cost benefit*"[All Fields] OR "social return on investment*"[All Fields] OR "stated preference*"[All Fields] OR "trade-off"[All Fields] OR "travel cost model*"[All Fields] OR "willingness to pay"[All Fields])) NOT ("Editorial"[Publication Type] OR "Comment"[Publication Type] OR "Clinical Trial Protocol"[Publication Type] OR "edit*"[Title] OR "comment*"[Title] OR "protocol*"[Title])) AND 2020/01/01:3000/12/31[Date - Create]) AND (english[Filter]) | | | |
