# Supplementary material for: The health economics of social prescribing: systematic review of the international evidence
Source: Front Public Health. 2026 Jan 28;14:1753435. doi: 10.3389/fpubh.2026.1753435 (PMC12891220; doi:10.3389/fpubh.2026.1753435)
Supplement: Supplementary file 3 [file Supplementary_file_3.docx]

## Supplementary File 3 – Quality appraisal tables

## Table 3.1 Quality appraisal of the randomized controlled studies (Barker et al. 2023)

| Citation | Q1. Was true randomization used for assignment of participants to treatment groups? | Q2. Was allocation to treatment groups concealed? | Q3. Were treatment groups similar at the baseline? | Q4. Were participants blind to treatment assignment? | Q5. Were those delivering treatment blind to treatment assignment? | Q6. Were treatment groups treated identically other than the intervention of interest? | Q7. Were outcomes assessors blind to treatment assignment? | Q8. Were outcomes measured in the same way for treatment groups? | Q9. Were outcomes measured in a reliable way | Q10. Was follow up complete and if not, were differences between groups in terms of their follow up adequately described and analyzed? | Q11. Were participants analyzed in the groups to which they were randomized? | Q12. Was appropriate statistical analysis used? | Q13. Was the trial design appropriate and any deviations from the standard RCT design (individual randomization, parallel groups) accounted for in the conduct and analysis of the trial? | Quality appraisal rating |
| --- | --- | --- | --- | --- | --- | --- | --- | --- | --- | --- | --- | --- | --- | --- |
| Clifford et al (2024) | Yes | Yes | Yes | Yes | Yes | Yes | Unclear | Yes | Yes | Yes | Yes | Yes | Yes | 12/13  High |
| Coulton et al (2015) | Yes | Yes | Yes | No | No | Yes | Yes | Yes | Yes | Yes | Yes | Yes | Yes | 11/13  High |
| Deidda et al (2022) | Yes | Yes | Yes | Unclear | Unclear | No | Unclear | Yes | Yes | Unclear | Yes | Yes | Yes | 8/13  Moderate |
| Ellis-Hill et al (2019) | Yes | Yes | Yes | No | No | Yes | Yes | Yes | Yes | Yes | Yes | Yes | Yes | 11/13 High |
| Tew et al (2024) | Yes | No | Unclear | No | No | Yes | No | Yes | Yes | Yes | Yes | Yes | Yes | 8/13  Moderate |

Note: Score between 1 and 5 is low quality; score between 6 and 9 is moderate quality; score between 10 and 13 is high quality.

## Table 3.2 Quality appraisal of the mixed methods studies (Munn 2021)

| Citation | 5.1 Is there an adequate rationale for using a mixed methods design to address the research question? | 5.2 Are the different components of the study effectively integrated to answer the research question? | 5.3 Are the outputs of the integration of qualitative and quantitative components adequately interpreted? | 5.4 Are divergences and inconsistencies between quantitative and qualitative results adequately addressed? | 5.5 Do the different components of the study adhere to the quality criteria of each tradition of the methods involved? | Quality appraisal rating |
| --- | --- | --- | --- | --- | --- | --- |
| Foster et al (2021) | Yes | Yes | Yes | Yes | Yes | 5/5 High |
| Gandy et al (2017) | Yes | Yes | Yes | No | Yes | 4/5 Moderate |
| Hartfiel et al (2023) | No | Yes | No | No | No | 1/5 Low |
| Jones et al (2020) | Yes | Yes | Yes | No | No | 3/5 Moderate |
| Lynch et al (2025) | Yes | Yes | No | Yes | Yes | 4/5 Moderate |
| Makanjuola et al (2022) | Yes | Yes | Yes | Yes | Yes | 5/5 High |
| Makanjuola et al (2023) | Yes | Yes | Yes | Yes | Yes | 5/5  High |
| Makanjuola et al (2025) | Yes | Yes | Yes | Yes | Yes | 5/5  High |
| Moffatt et al 2023 | Yes | Yes | Yes | Yes | Yes | 5/5  High |
| Skinner et al (2023) | No | No | Yes | No | No | 1/5 Low |
| Whiteley et al 2024 | Yes | Yes | Yes | Yes | Yes | 5/5  High |
| (Willis et al 2018) | No | No | Yes | No | No | 1/5 Low |

Note: Score between 1 and 2 is low quality; score between 3 and 4 is moderate quality, score of 5 is high quality.

## Table 3.3 Quality appraisal of the quasi-experimental study (Barker et al. 2024)

| Citation | 1. Is it clear in the study what is the “cause” and what is the “effect” (ie, there is no confusion about which variable comes first)? | 2. Was there a control group? | 3. Were participants included in any comparisons similar? | 4. Were the participants included in any comparisons receiving similar treatment/care, other than the exposure or intervention of interest? | 5. Were there multiple measurements of the outcome, both pre and post the intervention/exposure? | 6. Were the outcomes of participants included in any comparisons measured in the same way? | 7. Were outcomes measured in a reliable way? | 8. Was follow-up complete and, if not, were differences between groups in terms of their follow-up adequately described and analyzed? | 9.Was appropriate statistical analysis used? | Quality appraisal rating |
| --- | --- | --- | --- | --- | --- | --- | --- | --- | --- | --- |
| Galbraith et al (2022) | Yes | Yes | Yes | Yes | Yes | Yes | Yes | Yes | Yes | 9/9 High |

**Note:** Score between 1 and 3 is low quality; score between 4 and 6 is moderate quality; score between 7 and 9 is high quality.
